# Clinical significance of Janus Kinase inhibitor selectivity

**DOI:** 10.1093/rheumatology/kez002

**Published:** 2019-01-28

**Authors:** Ernest H Choy

**Affiliations:** CREATE Centre, Section of Rheumatology, Division of Infection and Immunity, Cardiff University School of Medicine, Cardiff, UK

Rheumatology 2019;58:953–62.

doi:10.1093/rheumatology/key339

In Table 3, the entry in the ‘DVT/PE’ row, first column ‘Tofacitinib (JAK, JAK3)’ has been replaced with ‘NR’ as it states later in the paper that this is not reported.

